# {5,5′-Bis(methoxy­carbonyl­meth­oxy)-2,2′-[ethane-1,2-diylbis(nitrilo­methyl­idyne)]­diphenolato}copper(II)

**DOI:** 10.1107/S1600536808033084

**Published:** 2008-10-18

**Authors:** Zhi-Hui Wang, Jian-Fang Ma, Hua Wu, Hai-Yan Liu

**Affiliations:** a Department of Chemistry, Northeast Normal University, Changchun 130024, People’s Republic of China

## Abstract

The title compound, [Cu(C_22_H_22_N_2_O_8_)], is a tetra­dentate Schiff base complex. The Cu^II^ ion has a nearly square-planar geometry, being coordinated by two N atoms and two O atoms. The two chemically equivalent halves of the mol­ecule are crystallographically independent. One of the carboxylic acid methyl ester units is located in the main plane of the mol­ecule and the other is rotated by 65.3 (5)° with respect to this unit. In the crystal structure, there are π–π stacking inter­actions between adjacent six-membered chelate rings, with centroid-to-centroid distances of 3.602 (2) Å.

## Related literature

For general background, see: Paschke *et al.* (2002 ▶); Blake *et al.* (1995 ▶). For related structures, see: Bbadbhade & Srinivas (1993 ▶). Shamim *et al.* (1988 ▶) report the synthesis of the precursor of the organic ligand.
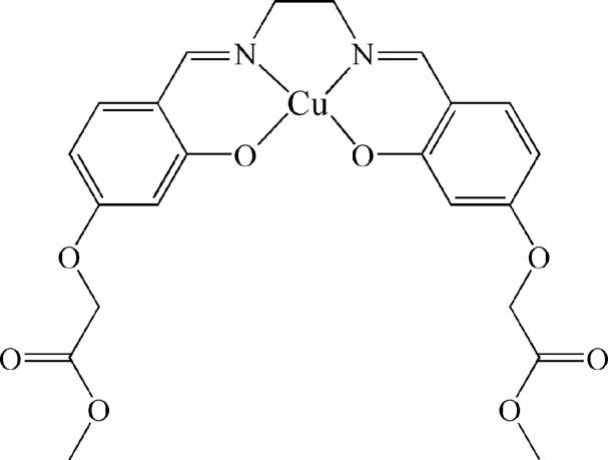


## Experimental

### 

#### Crystal data

[Cu(C_22_H_22_N_2_O_8_)]
*M* *_r_* = 505.96
Triclinic, 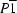

*a* = 9.668 (1) Å
*b* = 10.012 (1) Å
*c* = 11.763 (2) Å
α = 85.251 (2)°
β = 80.381 (2)°
γ = 75.383 (2)°
*V* = 1085.3 (2) Å^3^
*Z* = 2
Mo *K*α radiation
μ = 1.06 mm^−1^
*T* = 293 (2) K
0.40 × 0.30 × 0.25 mm

#### Data collection

Bruker APEX CCD area-detector diffractometer
Absorption correction: multi-scan (*SADABS*; Sheldrick, 1996 ▶) *T*_min_ = 0.677, *T*_max_ = 0.778
6326 measured reflections
4482 independent reflections
3096 reflections with *I* > 2σ(*I*)
*R*_int_ = 0.018

#### Refinement

*R*[*F* ^2^ > 2σ(*F* ^2^)] = 0.053
*wR*(*F* ^2^) = 0.124
*S* = 1.03
4482 reflections
298 parameters
H-atom parameters constrained
Δρ_max_ = 0.47 e Å^−3^
Δρ_min_ = −0.32 e Å^−3^

### 

Data collection: *SMART* (Bruker, 1997 ▶); cell refinement: *SAINT* (Bruker, 1999 ▶); data reduction: *SAINT*; program(s) used to solve structure: *SHELXS97* (Sheldrick, 2008 ▶); program(s) used to refine structure: *SHELXL97* (Sheldrick, 2008 ▶); molecular graphics: *SHELXTL-Plus* (Sheldrick, 2008 ▶); software used to prepare material for publication: *SHELXL97*.

## Supplementary Material

Crystal structure: contains datablocks global, I. DOI: 10.1107/S1600536808033084/zl2141sup1.cif

Structure factors: contains datablocks I. DOI: 10.1107/S1600536808033084/zl2141Isup2.hkl

Additional supplementary materials:  crystallographic information; 3D view; checkCIF report

## Figures and Tables

**Fig. 1.**
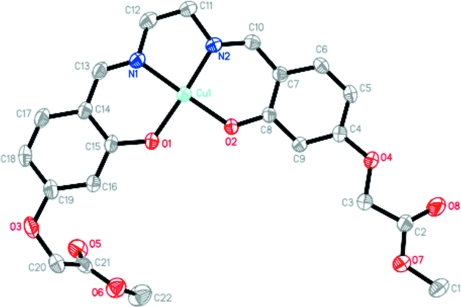
A view of the molecule of (I). Displacement ellipsoids are drawn at the 30% probability level. H atoms have been omitted for clarity.

**Fig. 2.**
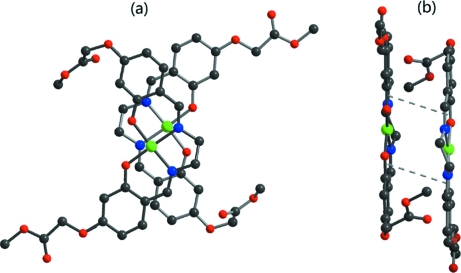
(a) Top and (b) side view of the title compound , showing π–π stacking and the formation of π-stacked dimers. For clarity, H atoms are omitted.

## supplementary crystallographic information

### Comment

There is a great interest in the use of Schiff base metal complexes as symmetrical and unsymmetrical liquid crystal compounds, because of their ready chemical modifiability (Paschke *et al.*, 2002). Substitution around the aromatic rings can drastically influence the structures and the properties of liquid crystal compounds. Many symmetrically substituted Salen-copper complexes reported are characterized by high melting and clearing temperatures, whereby detailed investigation is difficult because of decomposition after entering the S_A_ phase (Blake *et al.*, 1995). This work was incomplete in lacking direct structural evidence and the absence of ways to lower the melt temperatures. Afterwards, a series of asymmetrically substituted Salen-copper(II) complexes and ways of decreasing the melting temperatures by lateral and unsymmetrical substitution were reported (Paschke *et al.*, 2002). To further widen the scope of application of such compounds, we synthesized a new salen copper(II) compound and its structure is described in this paper.

As shown in Fig. 1, the title compound CuC_22_H_22_N_2_O_8_ is a tetradentate salen Schiff base complex. The Cu^II^ ion has a nearly square-planar geometry, being coordinated by two N atoms and two O atoms from the Schiff base ligand consisting of two Cu—O bonds [Cu(1)—O(1) = 1.897 (3) Å and Cu(1)—O(2) = 1.894 (2) Å], and two Cu—N bonds [Cu(1)—N(1) = 1.941 (3) Å and Cu(1)—N(2) = 1.922 (3) Å]. These bond distances are within the normal range observed in similar complexes (Bbadbhade & Srinivas, 1993). The two chemically equivalent halves of the molecule are crystallographically independent. One of the carboxylic acid methyl ester units is located in the main plane of the molecule, the other is rotated by 65.3 (5)° with respect to this unit.

As shown in Fig. 2, there are π–π stacking interactions between adjacent six-membered chelate rings, with a centroid–centroid distance of 3.602 (2) Å [symmetry code: -*x*, -*y*, 1 - *z*], which leads to the formation of π-stacked dimers of the title complex.

### Experimental

The first step is the preparation of 2-hydroxy-4-[(carboxymethyl)oxy]benzaldehyde methyl ester according to the reported procedure (Shamim *et al.*, 1988). The second step is the preparation of the ligand C_22_H_24_N_2_O_8_ (H_2_L). The white powder obtained in the first step (2.10 g, 10 mmol) was dissolved in methanol (40 ml), and ethylenediamine (0.30 g, 5 mmol) was added dropwise. The solution was stirred for 1 h, the yellow precipitation was collected, washed with ethanol, and then dried in a vacuum desiccator. The resulting yellow precipitate was H_2_L (1.55 g, 3.5 mmol, yield 70%).

H_2_L (0.022 g, 0.05 mmol) then dissolved in CHCl_3_ (20 ml), was stirred at room temperature and was added to the solution of CuNO_3_ (0.012 g, 0.05 mmol) in ethanol (20 ml). The mixture was stirred for 1 h and then the resulting solution was filtered and left in a dark place to slowly evaporate. Brown single crystals were obtained after several days (0.027 g, 0.04 mmol, yeild 80%). IR (cm^-1^, KBr): ν(C═N), 1606vs; ν(C═O), 1758vs; ν(C—OMe), 1213vs; ν(Ar—O), 1278vs.

### Refinement

All H-atoms bound to carbon were refined using a riding model with d(C—H) = 0.93 Å, *U*_iso_ = 1.2*U*_eq_ (C) for aromatic, 0.97 Å, *U*_iso_ = 1.2*U*_eq_ (C) for CH_2_, and 0.96 Å, *U*_iso_ = 1.5*U*_eq_ (C) for CH_3_ atoms.

### Crystal data

**Table:**

[Cu(C_22_H_22_N_2_O_8_)]	*Z* = 2
*M**_r_* = 505.96	*F*(000) = 522
Triclinic, *P*1	*D*_x_ = 1.548 Mg m^−^^3^
Hall symbol: -P 1	Mo *K*α radiation, λ = 0.71069 Å
*a* = 9.668 (1) Å	Cell parameters from 4482 reflections
*b* = 10.012 (1) Å	θ = 1.8–26.7°
*c* = 11.763 (2) Å	µ = 1.06 mm^−^^1^
α = 85.251 (2)°	*T* = 293 K
β = 80.381 (2)°	Block, brown
γ = 75.383 (2)°	0.40 × 0.30 × 0.25 mm
*V* = 1085.3 (2) Å^3^	

### Data collection

**Table:**

Bruker APEX CCD area-detector diffractometer	4482 independent reflections
Radiation source: fine-focus sealed tube	3096 reflections with *I* > 2σ(*I*)
graphite	*R*_int_ = 0.018
ω scans	θ_max_ = 26.7°, θ_min_ = 1.8°
Absorption correction: multi-scan (SADABS; Sheldrick, 1996)	*h* = −9→12
*T*_min_ = 0.677, *T*_max_ = 0.778	*k* = −9→12
6326 measured reflections	*l* = −13→14

### Refinement

**Table:**

Refinement on *F*^2^	Primary atom site location: structure-invariant direct methods
Least-squares matrix: full	Secondary atom site location: difference Fourier map
*R*[*F*^2^ > 2σ(*F*^2^)] = 0.053	Hydrogen site location: inferred from neighbouring sites
*wR*(*F*^2^) = 0.124	H-atom parameters constrained
*S* = 1.03	*w* = 1/[σ^2^(*F*_o_^2^) + (0.0435*P*)^2^ + 0.7616*P*] where *P* = (*F*_o_^2^ + 2*F*_c_^2^)/3
4482 reflections	(Δ/σ)_max_ < 0.001
298 parameters	Δρ_max_ = 0.47 e Å^−^^3^
0 restraints	Δρ_min_ = −0.32 e Å^−^^3^

### Special details

**Table:**

Geometry. All e.s.d.'s (except the e.s.d. in the dihedral angle between two l.s. planes) are estimated using the full covariance matrix. The cell e.s.d.'s are taken into account individually in the estimation of e.s.d.'s in distances, angles and torsion angles; correlations between e.s.d.'s in cell parameters are only used when they are defined by crystal symmetry. An approximate (isotropic) treatment of cell e.s.d.'s is used for estimating e.s.d.'s involving l.s. planes.
Refinement. Refinement of *F*^2^ against ALL reflections. The weighted *R*-factor *wR* and goodness of fit *S* are based on *F*^2^, conventional *R*-factors *R* are based on *F*, with *F* set to zero for negative *F*^2^. The threshold expression of *F*^2^ > σ(*F*^2^) is used only for calculating *R*-factors(gt) *etc*. and is not relevant to the choice of reflections for refinement. *R*-factors based on *F*^2^ are statistically about twice as large as those based on *F*, and *R*- factors based on ALL data will be even larger.

### Fractional atomic coordinates and isotropic or equivalent isotropic displacement parameters (Å^2^)

**Table:**

	*x*	*y*	*z*	*U*_iso_*/*U*_eq_	
Cu1	−0.02639 (5)	0.15738 (5)	0.40718 (4)	0.04568 (17)	
C1	0.8714 (5)	0.5674 (5)	0.3845 (4)	0.0811 (15)	
H1A	0.8657	0.6225	0.4488	0.122*	
H1B	0.8845	0.6213	0.3139	0.122*	
H1C	0.9518	0.4882	0.3850	0.122*	
C2	0.7312 (5)	0.4448 (4)	0.3101 (4)	0.0581 (11)	
C3	0.5930 (4)	0.3988 (4)	0.3310 (3)	0.0519 (10)	
H3A	0.5849	0.3465	0.4039	0.062*	
H3B	0.5108	0.4782	0.3334	0.062*	
C4	0.4834 (4)	0.2571 (4)	0.2380 (3)	0.0528 (10)	
C5	0.4966 (4)	0.1810 (5)	0.1410 (3)	0.0578 (11)	
H5	0.5777	0.1718	0.0845	0.069*	
C6	0.3889 (4)	0.1208 (4)	0.1310 (3)	0.0558 (10)	
H6	0.3973	0.0705	0.0661	0.067*	
C7	0.2642 (4)	0.1313 (4)	0.2149 (3)	0.0466 (9)	
C8	0.2522 (4)	0.2083 (4)	0.3138 (3)	0.0447 (9)	
C9	0.3649 (4)	0.2707 (4)	0.3234 (3)	0.0499 (9)	
H9	0.3593	0.3212	0.3877	0.060*	
C10	0.1556 (4)	0.0667 (4)	0.1949 (3)	0.0546 (10)	
H10	0.1739	0.0167	0.1286	0.066*	
C11	−0.0766 (5)	0.0142 (6)	0.2262 (4)	0.0791 (15)	
H11A	−0.1344	0.0822	0.1785	0.095*	
H11B	−0.0307	−0.0662	0.1809	0.095*	
C12	−0.1694 (5)	−0.0249 (5)	0.3272 (4)	0.0785 (15)	
H12A	−0.1265	−0.1178	0.3546	0.094*	
H12B	−0.2629	−0.0241	0.3069	0.094*	
C13	−0.2968 (4)	0.0871 (4)	0.4993 (4)	0.0567 (11)	
H13	−0.3664	0.0395	0.4938	0.068*	
C14	−0.3203 (4)	0.1694 (4)	0.5965 (3)	0.0484 (9)	
C15	−0.2202 (4)	0.2436 (4)	0.6176 (3)	0.0461 (9)	
C16	−0.2502 (4)	0.3153 (4)	0.7201 (3)	0.0501 (10)	
H16	−0.1866	0.3655	0.7347	0.060*	
C17	−0.4452 (4)	0.1743 (5)	0.6791 (4)	0.0609 (11)	
H17	−0.5127	0.1285	0.6649	0.073*	
C18	−0.4712 (4)	0.2421 (5)	0.7772 (4)	0.0628 (12)	
H18	−0.5545	0.2420	0.8297	0.075*	
C19	−0.3721 (4)	0.3127 (4)	0.7997 (3)	0.0548 (10)	
C20	−0.3016 (4)	0.4378 (5)	0.9347 (4)	0.0638 (12)	
H20A	−0.3435	0.4885	1.0040	0.077*	
H20B	−0.2760	0.5030	0.8741	0.077*	
C21	−0.1672 (5)	0.3315 (5)	0.9570 (3)	0.0571 (11)	
C22	0.0779 (5)	0.3088 (6)	0.9761 (5)	0.1005 (19)	
H22A	0.1489	0.3625	0.9632	0.151*	
H22B	0.1110	0.2290	0.9299	0.151*	
H22C	0.0634	0.2799	1.0562	0.151*	
N1	−0.1881 (3)	0.0725 (3)	0.4185 (3)	0.0542 (8)	
N2	0.0342 (3)	0.0715 (3)	0.2610 (3)	0.0540 (8)	
O1	−0.1002 (3)	0.2473 (3)	0.5482 (2)	0.0537 (7)	
O2	0.1423 (3)	0.2237 (3)	0.3977 (2)	0.0534 (7)	
O3	−0.4053 (3)	0.3772 (3)	0.9017 (2)	0.0682 (8)	
O4	0.5962 (3)	0.3158 (3)	0.2393 (2)	0.0674 (8)	
O5	−0.1593 (3)	0.2113 (3)	0.9815 (3)	0.0723 (9)	
O6	−0.0568 (3)	0.3915 (3)	0.9447 (3)	0.0736 (9)	
O7	0.7385 (3)	0.5219 (3)	0.3929 (2)	0.0650 (8)	
O8	0.8223 (4)	0.4170 (5)	0.2289 (4)	0.1257 (18)	

### Atomic displacement parameters (Å^2^)

**Table:**

	*U*^11^	*U*^22^	*U*^33^	*U*^12^	*U*^13^	*U*^23^
Cu1	0.0420 (3)	0.0513 (3)	0.0476 (3)	−0.0201 (2)	−0.0019 (2)	−0.0069 (2)
C1	0.071 (3)	0.092 (4)	0.095 (4)	−0.043 (3)	−0.011 (3)	−0.020 (3)
C2	0.058 (3)	0.058 (3)	0.062 (3)	−0.023 (2)	0.001 (2)	−0.009 (2)
C3	0.051 (2)	0.059 (3)	0.049 (2)	−0.0212 (19)	−0.0031 (18)	−0.003 (2)
C4	0.052 (2)	0.061 (3)	0.048 (2)	−0.024 (2)	−0.0011 (19)	−0.001 (2)
C5	0.050 (2)	0.075 (3)	0.048 (2)	−0.023 (2)	0.0092 (19)	−0.008 (2)
C6	0.056 (2)	0.066 (3)	0.044 (2)	−0.018 (2)	0.0061 (19)	−0.016 (2)
C7	0.049 (2)	0.044 (2)	0.046 (2)	−0.0125 (17)	−0.0030 (18)	−0.0057 (18)
C8	0.042 (2)	0.048 (2)	0.045 (2)	−0.0152 (17)	0.0003 (17)	−0.0038 (17)
C9	0.051 (2)	0.057 (2)	0.047 (2)	−0.0233 (19)	0.0004 (18)	−0.0109 (19)
C10	0.059 (2)	0.060 (3)	0.049 (2)	−0.022 (2)	−0.001 (2)	−0.016 (2)
C11	0.073 (3)	0.113 (4)	0.069 (3)	−0.053 (3)	0.000 (2)	−0.033 (3)
C12	0.073 (3)	0.093 (4)	0.086 (3)	−0.050 (3)	−0.005 (3)	−0.025 (3)
C13	0.046 (2)	0.065 (3)	0.066 (3)	−0.029 (2)	−0.007 (2)	0.002 (2)
C14	0.039 (2)	0.055 (2)	0.053 (2)	−0.0189 (18)	−0.0059 (18)	0.0068 (19)
C15	0.0350 (19)	0.055 (2)	0.047 (2)	−0.0136 (17)	−0.0037 (17)	0.0067 (19)
C16	0.040 (2)	0.062 (3)	0.049 (2)	−0.0179 (18)	−0.0001 (17)	−0.0035 (19)
C17	0.044 (2)	0.073 (3)	0.071 (3)	−0.029 (2)	−0.004 (2)	0.002 (2)
C18	0.041 (2)	0.077 (3)	0.066 (3)	−0.018 (2)	0.011 (2)	0.003 (2)
C19	0.041 (2)	0.064 (3)	0.052 (2)	−0.0060 (19)	0.0019 (19)	0.000 (2)
C20	0.053 (2)	0.072 (3)	0.061 (3)	−0.011 (2)	0.009 (2)	−0.018 (2)
C21	0.065 (3)	0.065 (3)	0.038 (2)	−0.014 (2)	0.0038 (19)	−0.010 (2)
C22	0.075 (4)	0.095 (4)	0.136 (5)	−0.007 (3)	−0.044 (3)	−0.012 (4)
N1	0.0485 (19)	0.060 (2)	0.061 (2)	−0.0244 (16)	−0.0032 (17)	−0.0137 (17)
N2	0.054 (2)	0.063 (2)	0.053 (2)	−0.0263 (17)	−0.0062 (16)	−0.0137 (17)
O1	0.0457 (14)	0.0697 (19)	0.0502 (15)	−0.0286 (13)	0.0075 (12)	−0.0129 (14)
O2	0.0483 (15)	0.0673 (18)	0.0489 (15)	−0.0277 (13)	0.0086 (12)	−0.0172 (13)
O3	0.0498 (17)	0.088 (2)	0.0567 (18)	−0.0110 (16)	0.0147 (14)	−0.0127 (16)
O4	0.0584 (17)	0.093 (2)	0.0611 (18)	−0.0453 (17)	0.0108 (14)	−0.0208 (16)
O5	0.087 (2)	0.063 (2)	0.065 (2)	−0.0197 (17)	−0.0041 (16)	−0.0023 (16)
O6	0.066 (2)	0.070 (2)	0.086 (2)	−0.0150 (17)	−0.0160 (17)	−0.0054 (18)
O7	0.0600 (18)	0.078 (2)	0.0647 (19)	−0.0299 (16)	−0.0067 (15)	−0.0144 (16)
O8	0.093 (3)	0.181 (4)	0.126 (3)	−0.091 (3)	0.052 (3)	−0.097 (3)

### Geometric parameters (Å, °)

**Table:**

Cu1—O2	1.894 (2)	C11—H11A	0.9700
Cu1—O1	1.897 (3)	C11—H11B	0.9700
Cu1—N2	1.922 (3)	C12—N1	1.469 (5)
Cu1—N1	1.941 (3)	C12—H12A	0.9700
C1—O7	1.454 (5)	C12—H12B	0.9700
C1—H1A	0.9600	C13—N1	1.281 (5)
C1—H1B	0.9600	C13—C14	1.419 (6)
C1—H1C	0.9600	C13—H13	0.9300
C2—O8	1.186 (5)	C14—C17	1.411 (5)
C2—O7	1.313 (5)	C14—C15	1.422 (5)
C2—C3	1.497 (5)	C15—O1	1.309 (4)
C3—O4	1.407 (4)	C15—C16	1.405 (5)
C3—H3A	0.9700	C16—C19	1.381 (5)
C3—H3B	0.9700	C16—H16	0.9300
C4—O4	1.366 (4)	C17—C18	1.342 (6)
C4—C9	1.379 (5)	C17—H17	0.9300
C4—C5	1.394 (5)	C18—C19	1.395 (6)
C5—C6	1.353 (5)	C18—H18	0.9300
C5—H5	0.9300	C19—O3	1.362 (5)
C6—C7	1.412 (5)	C20—O3	1.415 (5)
C6—H6	0.9300	C20—C21	1.504 (6)
C7—C8	1.421 (5)	C20—H20A	0.9700
C7—C10	1.423 (5)	C20—H20B	0.9700
C8—O2	1.310 (4)	C21—O5	1.200 (5)
C8—C9	1.410 (5)	C21—O6	1.333 (5)
C9—H9	0.9300	C22—O6	1.444 (5)
C10—N2	1.287 (5)	C22—H22A	0.9600
C10—H10	0.9300	C22—H22B	0.9600
C11—C12	1.453 (6)	C22—H22C	0.9600
C11—N2	1.463 (5)		
			
O2—Cu1—O1	89.23 (10)	C11—C12—H12B	109.7
O2—Cu1—N2	93.86 (12)	N1—C12—H12B	109.7
O1—Cu1—N2	175.69 (13)	H12A—C12—H12B	108.2
O2—Cu1—N1	174.74 (13)	N1—C13—C14	125.7 (4)
O1—Cu1—N1	92.91 (12)	N1—C13—H13	117.2
N2—Cu1—N1	84.26 (13)	C14—C13—H13	117.2
O7—C1—H1A	109.5	C17—C14—C13	119.1 (4)
O7—C1—H1B	109.5	C17—C14—C15	117.9 (4)
H1A—C1—H1B	109.5	C13—C14—C15	123.0 (3)
O7—C1—H1C	109.5	O1—C15—C16	117.8 (3)
H1A—C1—H1C	109.5	O1—C15—C14	124.0 (4)
H1B—C1—H1C	109.5	C16—C15—C14	118.2 (3)
O8—C2—O7	123.9 (4)	C19—C16—C15	121.4 (4)
O8—C2—C3	124.7 (4)	C19—C16—H16	119.3
O7—C2—C3	111.4 (4)	C15—C16—H16	119.3
O4—C3—C2	107.1 (3)	C18—C17—C14	123.0 (4)
O4—C3—H3A	110.3	C18—C17—H17	118.5
C2—C3—H3A	110.3	C14—C17—H17	118.5
O4—C3—H3B	110.3	C17—C18—C19	119.4 (4)
C2—C3—H3B	110.3	C17—C18—H18	120.3
H3A—C3—H3B	108.5	C19—C18—H18	120.3
O4—C4—C9	124.2 (4)	O3—C19—C16	124.2 (4)
O4—C4—C5	114.2 (3)	O3—C19—C18	115.8 (3)
C9—C4—C5	121.7 (4)	C16—C19—C18	120.0 (4)
C6—C5—C4	118.6 (4)	O3—C20—C21	112.0 (4)
C6—C5—H5	120.7	O3—C20—H20A	109.2
C4—C5—H5	120.7	C21—C20—H20A	109.2
C5—C6—C7	122.7 (4)	O3—C20—H20B	109.2
C5—C6—H6	118.6	C21—C20—H20B	109.2
C7—C6—H6	118.6	H20A—C20—H20B	107.9
C6—C7—C8	118.3 (3)	O5—C21—O6	125.0 (4)
C6—C7—C10	118.4 (4)	O5—C21—C20	125.8 (4)
C8—C7—C10	123.3 (3)	O6—C21—C20	109.2 (4)
O2—C8—C9	117.8 (3)	O6—C22—H22A	109.5
O2—C8—C7	123.6 (3)	O6—C22—H22B	109.5
C9—C8—C7	118.5 (3)	H22A—C22—H22B	109.5
C4—C9—C8	120.2 (4)	O6—C22—H22C	109.5
C4—C9—H9	119.9	H22A—C22—H22C	109.5
C8—C9—H9	119.9	H22B—C22—H22C	109.5
N2—C10—C7	125.5 (4)	C13—N1—C12	120.7 (3)
N2—C10—H10	117.2	C13—N1—Cu1	126.4 (3)
C7—C10—H10	117.2	C12—N1—Cu1	112.7 (2)
C12—C11—N2	110.4 (4)	C10—N2—C11	121.1 (3)
C12—C11—H11A	109.6	C10—N2—Cu1	125.9 (3)
N2—C11—H11A	109.6	C11—N2—Cu1	112.9 (3)
C12—C11—H11B	109.6	C15—O1—Cu1	128.0 (2)
N2—C11—H11B	109.6	C8—O2—Cu1	127.6 (2)
H11A—C11—H11B	108.1	C19—O3—C20	118.2 (3)
C11—C12—N1	109.6 (4)	C4—O4—C3	119.5 (3)
C11—C12—H12A	109.7	C21—O6—C22	117.1 (4)
N1—C12—H12A	109.7	C2—O7—C1	116.2 (3)
			
O8—C2—C3—O4	1.7 (7)	C14—C13—N1—C12	174.8 (4)
O7—C2—C3—O4	−179.1 (3)	C14—C13—N1—Cu1	0.4 (6)
O4—C4—C5—C6	−178.9 (4)	C11—C12—N1—C13	158.0 (4)
C9—C4—C5—C6	0.7 (7)	C11—C12—N1—Cu1	−26.8 (5)
C4—C5—C6—C7	−0.4 (7)	O1—Cu1—N1—C13	0.8 (4)
C5—C6—C7—C8	−0.1 (6)	N2—Cu1—N1—C13	−175.9 (4)
C5—C6—C7—C10	178.6 (4)	O1—Cu1—N1—C12	−174.0 (3)
C6—C7—C8—O2	−179.2 (4)	N2—Cu1—N1—C12	9.3 (3)
C10—C7—C8—O2	2.2 (6)	C7—C10—N2—C11	173.2 (4)
C6—C7—C8—C9	0.1 (6)	C7—C10—N2—Cu1	−4.0 (6)
C10—C7—C8—C9	−178.5 (4)	C12—C11—N2—C10	154.5 (4)
O4—C4—C9—C8	178.9 (4)	C12—C11—N2—Cu1	−28.0 (5)
C5—C4—C9—C8	−0.7 (6)	O2—Cu1—N2—C10	2.8 (4)
O2—C8—C9—C4	179.6 (4)	N1—Cu1—N2—C10	−172.3 (4)
C7—C8—C9—C4	0.3 (6)	O2—Cu1—N2—C11	−174.6 (3)
C6—C7—C10—N2	−177.2 (4)	N1—Cu1—N2—C11	10.3 (3)
C8—C7—C10—N2	1.4 (7)	C16—C15—O1—Cu1	177.8 (3)
N2—C11—C12—N1	34.7 (6)	C14—C15—O1—Cu1	−0.9 (5)
N1—C13—C14—C17	−179.5 (4)	O2—Cu1—O1—C15	−175.7 (3)
N1—C13—C14—C15	−2.2 (7)	N1—Cu1—O1—C15	−0.6 (3)
C17—C14—C15—O1	179.8 (3)	C9—C8—O2—Cu1	178.0 (3)
C13—C14—C15—O1	2.5 (6)	C7—C8—O2—Cu1	−2.7 (5)
C17—C14—C15—C16	1.1 (5)	O1—Cu1—O2—C8	−176.5 (3)
C13—C14—C15—C16	−176.2 (4)	N2—Cu1—O2—C8	0.4 (3)
O1—C15—C16—C19	−177.8 (3)	C16—C19—O3—C20	−7.3 (6)
C14—C15—C16—C19	1.0 (6)	C18—C19—O3—C20	173.4 (4)
C13—C14—C17—C18	175.5 (4)	C21—C20—O3—C19	−65.3 (5)
C15—C14—C17—C18	−1.9 (6)	C9—C4—O4—C3	−1.7 (6)
C14—C17—C18—C19	0.6 (7)	C5—C4—O4—C3	177.9 (4)
C15—C16—C19—O3	178.4 (4)	C2—C3—O4—C4	178.6 (3)
C15—C16—C19—C18	−2.4 (6)	O5—C21—O6—C22	−7.4 (6)
C17—C18—C19—O3	−179.1 (4)	C20—C21—O6—C22	172.9 (4)
C17—C18—C19—C16	1.5 (6)	O8—C2—O7—C1	−3.5 (7)
O3—C20—C21—O5	−22.9 (6)	C3—C2—O7—C1	177.3 (4)
O3—C20—C21—O6	156.8 (3)		
